# Differences in reproductive risk factors for breast cancer in middle-aged women in Marin County, California and a sociodemographically similar area of Northern California

**DOI:** 10.1186/1472-6874-9-6

**Published:** 2009-03-25

**Authors:** C Suzanne Lea, Nancy P Gordon, Lee Ann Prebil, Rochelle Ereman, Connie S Uratsu, Mark Powell

**Affiliations:** 1 Department of Public Health, Brody School of Medicine, East Carolina University, Greenville, NC 27834, USA; 2 Kaiser Permanente Division of Research, 2000 Broadway, Oakland, CA 94612, USA; 3 Marin County, Department of Health and Human Services, 20 North San Pedro Road, Suite 2002, San Rafael, CA 94903, USA

## Abstract

**Background:**

The Northern California county of Marin (MC) has historically had high breast cancer incidence rates. Because of MC's high socioeconomic status (SES) and racial homogeneity (non-Hispanic White), it has been difficult to assess whether these elevated rates result from a combination of established risk factors or other behavioral or environmental factors. This survey was designed to compare potential breast cancer risks and incidence rates for a sample of middle-aged MC women with those of a demographically similar population.

**Methods:**

A random sample of 1500 middle-aged female members of a large Northern California health plan, half from Marin County (MC) and half from a comparison area in East/Central Contra Costa County (ECCC), were mailed a survey covering family history, reproductive history, use of oral contraceptives (OC) and hormone replacement therapy (HRT), behavioral health risks, recency of breast screening, and demographic characteristics. Weighted data were used to compare prevalence of individual breast cancer risk factors and Gail scores. Age-adjusted cumulative breast cancer incidence rates (2000–2004) were also calculated for female health plan members aged 40–64 residing in the two geographic areas.

**Results:**

Survey response was 57.1% (n = 427) and 47.9% (n = 359) for MC and ECCC samples, respectively. Women in the two areas were similar in SES, race, obesity, exercise frequency, current smoking, ever use of OCs and HRT, age at onset of menarche, high mammography rates, family history of breast cancer, and Gail scores. However, MC women were significantly more likely than ECCC women to be former smokers (43.6% vs. 31.2%), have Ashkenazi Jewish heritage (12.8% vs. 7.1%), have no live births before age 30 (52.7% vs. 40.8%), and be nulliparous (29.2% vs. 15.4%), and less likely to never or rarely consume alcohol (34.4% vs. 41.9%). MC and ECCC women had comparable 2000–2004 invasive breast cancer incidence rates.

**Conclusion:**

The effects of reproductive risks factors, Ashkenazi Jewish heritage, smoking history, and alcohol consumption with regard to breast cancer risk in Marin County should be further evaluated. When possible, future comparisons of breast cancer incidence rates between regions should adjust for differences in income and education in addition to age and race/ethnicity, preferably by using a sociodemographically similar comparison group.

## Background

The San Francisco Bay Area (SFBA) has some of the highest breast cancer incidence rates in the United States [1]. Marin County, California, which lies north across the San Francisco Bay from San Francisco, California, had breast cancer rates of 176.6 per 100,000 among White non-Hispanic women between 1997–2001, 15% higher than those seen in California as a whole [2]. Previous epidemiologic studies have suggested that the high breast cancer incidence rates seen in the SFBA region parallel the high levels of established risk factors for this disease, including low parity, late age at first birth, high socioeconomic status (SES), and high alcohol consumption compared to other California counties [3-8]. However, data on the prevalence of traditional risk factors in a sample from Marin County compared to another county with similar SES have been lacking.

In response to the Marin community's concerns about the elevated breast cancer rate, Kaiser Permanente Medical Care Program, the largest health care provider in the area, and the Marin County Department of Health Services collaborated on a pilot study designed to compare prevalence of known breast cancer risk factors and breast cancer incidence among Kaiser Permanente health plan members in Marin County with those of health plan members in a defined geographic area of the SFBA that was sociodemographically similar to Marin County.

## Methods

All women in this study were sampled from the KPNC membership based on residential address and age (40–65). The Marin County (MC) group was a random sample of 750 female members who resided within Marin County. The comparison group was a random sample of 750 female members who resided in East/Central Contra Costa County (ECCC), which included the cities of Walnut Creek, Lafayette, Moraga, Orinda, Pleasant Hill, Concord, San Ramon, and Danville. The ECCC catchment area, which is geographically near MC but separated by the San Francisco Bay and the Oakland/Berkeley Hills, was selected because it was found to be demographically comparable to MC with regard to race/ethnicity (>80% non-Hispanic White), education (approximately 50% college graduates), and upper middle class income (>35% with a 1998 household income ≥$80,000) based on KPNC's 1999 Member Health Survey [9]. Based on data from the 2003 California Health Interview Survey [10], we estimate that approximately 28% of Marin County women and 38% of ECCC women in this age group were KPNC members in 2003. The sample size was chosen based on affordability and 80% power to detect a 10 percentage point difference in prevalence of individual breast cancer risk factors, assuming our anticipated 75% response rate among eligible women.

Women in both areas were sent a confidential "Women's Health Risk Factor Survey" questionnaire, accompanied by a cover letter from KPNC's Division of Research explaining that the study was being conducted to investigate the extent to which breast cancer rates in certain counties could be explained by differences in the percentages of women in those counties who have factors known to increase breast cancer risk. The survey questions were drawn from previous epidemiologic studies of breast cancer in California [3] or the KPNC Member Health Survey. The first mailing was sent in early November 2003, with non-respondents subsequently sent up to two additional survey mailings in February and March of 2004. The protocol for this study was approved by KPNC's Institutional Review Board for the Protection of Human Subjects.

The questionnaire collected information about established breast cancer risk factors, including demographics (race/ethnicity, education, income), breast health (history of breast cancer or breast biopsies), family history of breast cancer, reproductive history (age at onset and cessation of menstrual periods, number of pregnancies, parity, and age at first birth), use of birth control pills and hormone replacement therapy (HRT), and behavioral health risks, including cigarette smoking, height and weight to calculate body mass index (BMI), usual frequency of moderate or strenuous exercise per week, and average weekly alcohol consumption (based on number of days per week alcohol was consumed and number of drinks usually consumed on those days). Women were also asked to report the timing of their last mammogram. However, since screening mammography is not recommended to start until age 40, comparisons were restricted to age ≥41 to give the younger women at least 12 months to have had a first mammogram. A copy of the questionnaire can be found in Additional file 1.

Based on information about individual risk factors, a Gail risk score [11] was calculated for each respondent with no reported history of breast cancer using the National Cancer Institute's Breast Cancer Risk Assessment Tool. The Gail risk score is derived from a statistical model that estimates risk of invasive breast cancer for women who have had no previous invasive breast cancer and who have no evidence of breast cancer at the time of their screening mammogram [11-13]. Components of the model include: a woman's race, current age, age at menarche, age at first full-term pregnancy, number of first-degree relatives with breast cancer, number of breast biopsies and number of biopsies with atypical hyperplasia. Responses from the risk survey were entered into the score calculation website. A risk calculator available on the website generated the score to one decimal place and was recorded manually [14]. For women aged ≥35 with no history of breast cancer, Gail risk scores represent the estimated risk of being diagnosed with invasive breast cancer in the next five years compared to women of the same age and race/ethnicity from the general U.S. population [12]. A five-year Gail risk score ≥1.67% is considered high, but because the website-generated Gail risk scores went out to only one decimal place, we classified scores of ≥1.7% as high risk.

Analyses were performed using SAS (Statistical Analysis Software) v.9.1 procedures for analysis of data obtained from complex survey designs [15]. All analyses used respondent data weighted to reflect the actual age distribution (in 5-year intervals) of female Kaiser members aged 40–65 living in the MC and ECCC geographic areas from which the samples were drawn at the time of the survey. SAS Proc Surveyfreq for complex survey designs was used to calculate prevalence of risk factors with 95% confidence intervals and to calculate a chi-square statistic to determine the statistical significance of differences between the two groups. SAS Proc Surveyreg was used to determine whether differences in continuous variables (e.g., Gail risk score ≥1.70) were statistically significant.

Concurrent with the survey analyses, cumulative breast cancer incidence rates (2000–2004) were calculated for all KPNC female members aged 40 to 64 residing in MC and the ECCC comparison areas, defined by the same cluster of residence zip codes used to pick the survey samples. Cases of newly diagnosed, invasive breast cancer tumors [16] (ICD-O-3 primary site codes: C500–506, C508, C509 [17]) in the MC (n = 185) and ECCC (n = 384) geographic areas were identified from the KPNC Cancer Registry. Denominators for each geographic area were created from counts of female health plan members aged 40–64 active in June of each year, 2000–2004 (MC: n = 65,937; ECCC: n = 148,388 – cumulative for five years).

Incidence rates of all invasive breast cancers (SEER summary stage 1) for each study area were adjusted using the direct method to the age distribution of the 2000 US Census female population residing in that geographic area. Log-linear Poisson regression was used to estimate the relative risk, adjusted for age (ten 5-year age categories) and ethnicity. While KPNC did not have computerized race/ethnicity data on its membership, estimates based on KPNC's 2002 Member Health Survey [9] suggested that 90% of women aged 40–65 in the MC area were non-Hispanic White compared with 81% non-Hispanic White in the ECCC area. To take into account the difference in race/ethnic composition of the populations, race/ethnicity percents by 5-year age group were estimated for each geographic area using the US 2000 Census. Percents were adjusted by the proportion of KPNC females living in each Census tract within the MC or ECCC geographic area by 5-year age groups. In order to calculate incidence rates for the non-Hispanic White population, a subset of females with breast cancer diagnosed in 2000–2004 was selected from the KPNC Cancer Registry based on the race (White) and Hispanic origin (No) data fields. Cumulative incidence rates for each area were calculated using the count of cases in the numerator and multiplying the member count by the corresponding percent of non-Hispanic white females as the denominator. Poisson regression showed similar results for all races combined. All analyses were performed using SAS (v 9.1).

## Results

Response rates for the MC and ECCC samples were 57.1% (427/748) and 47.9% (359/749), respectively, after excluding persons for whom no forwarding address could be found. Interviews with random samples of non-respondents from both areas suggested that non-participation was primarily due to women being too busy with family and work responsibilities.

Table 1 displays the demographic characteristics of female respondents from the MC and ECCC groups. The MC and ECCC groups were similar in age at the time of the survey (mean ages 52.6 and 51.6 years, respectively), though the MC group had somewhat higher proportions of women at the upper end of the age range (p = .053). Both groups were predominantly (> 85%) non-Hispanic White, but the ECCC group had a significantly higher percentage of Asian women (4.2% vs. 10.8%, p < 0.0002). The MC group had a significantly higher percentage of women with Ashkenazi Jewish heritage (12.9% vs. 7.1%, p = 0.012). As intended by the sampling frame, both groups had similarly high levels of educational attainment (> 55% college graduate or post-graduate degrees) and household income (> 34% with incomes > $100,000). The two groups were comparable with respect to having first degree relatives with breast cancer. Both groups had similarly low percentages of women who had been diagnosed with breast cancer and similar proportions of women who reported one or more biopsies.

**Table 1:** Demographic Characteristics and Breast Cancer History of Female Kaiser Permanente Members Aged 40–65 in Marin County and East/Central Contra Costa County, California

	Marin		East/Central Contra Costa		
			
	Base No.	Percent (95% CI)	Base No.	Percent (95% CI)	p-value
**Age**	426		363		p < .053
40 – 45 years		20.0 (15.9 – 24.1)		22.8 (18.3 – 27.4)	
46 – 50 years		20.1 (16.4 – 23.9)		23.7 (19.3 – 28.2)	
51 – 55 years		23.8 (19.8 – 27.8)		23.1 (18.7 – 27.6)	
56 – 60 years		20.3 (16.4 – 24.2)		17.1 (13.1 – 21.1)	
61 – 65 years		15.8 (12.4 – 19.1)		13.2 (10.2 – 16.2)	
					
**Race/Ethnicity**	423		361		p = .007
Caucasian		88.3 (85.2 – 91.4)		84.3 (80.5 – 88.1)	
African American		1.2 (0.1 – 2.2)		0.9 (<0.1 – 1.7)	
Hispanic		4.9 (2.8 – 7.0)		3.7 (1.7 – 5.6)	
Asian		4.2 (2.2 – 6.1)		10.8 (7.6 – 14.1)	
Other		1.4 (0.3 – 2.5)		0.3 (<0.1 – 0.8)	
					
**Ashkenazi Jewish heritage**	416	12.8 (9.6 – 16.0)	357	7.1 (4.4 – 9.8)	p = .012
					
**Household income**	405		345		p = .14
≤ $35, 000		11.6 (8.4 – 14.7)		6.3 (3.8 – 8.9)	
> $35,000 to $50,000		10.2 (7.3 – 13.1)		9.9 (6.8 – 13.2)	
> $50,000 to $80,000		26.0 (21.7 – 30.3)		27.2 (22.5 – 32.0)	
> $80,000 to $100,000		17.6 (13.8 – 21.4)		17.2 (13.1 – 21.2)	
> $100,000		34.6 (29.9 – 39.3)		39.1 (33.9 – 44.5)	
					
**Education level**	425		363		p = .47
≤High school grad		6.4 (4.1 – 8.7)		6.1 (3.6 – 8.5)	
Some college/tech school		36.4 (31.8 – 41.0)		36.7 (31.7 – 41.8)	
College graduate		30.2 (25.8 – 34.7)		34.5 (29.6 – 39.5)	
Graduate degree		25.6 (21.4 – 29.8)		22.7 (18.2 – 27.1)	
					
**History of breast cancer**	425	5.0 (2.9 – 7.0)	363	4.6 (2.4 – 6.8)	p = .77
					
**First degree relatives with history of breast cancer**	426		363		
Daughter		1.1 (0 – 2.3)		0.6 (0 – 1.4)	p = .61
Mother		13.7 (10.2 – 17.3)		11.4 (7.9 – 14.9)	p = .44
Sister		3.6 (1.7 – 5.6)		4.4 (2.1 – 6.8)	p = .56
≥ 1 first degree female relative		14.8 (11.4 – 18.2)		13.6 (10.0 – 17.2)	p = .43
					
**≥1 biopsy to check for cancer**	425	21.8 (17.9 – 25.7)	363	17.2 (13.3 – 21.2)	p = .12

Table 2 shows a comparison of the MC and ECCC groups with regard to hormone use and reproductive risk factors accounting for differences in the age distribution between the two groups. The two groups did not differ significantly on age at onset of menarche or exogenous hormone use. The percentage of women who were post-menopausal was higher in the MC group (57.7% vs. 51.1%, p = .08), and the MC group had a significantly higher percentage of women with menopause due to natural (versus surgical) causes (47.8% vs. 36.2%, p = .002). Overall, MC women were significantly more likely than the ECCC women to be nulliparous (29.2% vs. 15.4%, p < 0.0001), significantly less likely to be multiparous (51.5% vs. 65.5%, p < .0011), and significantly less likely to have borne a child by the age of 30 (52.7% vs. 40.8%, p = .001), but they did not significantly differ with regard to breastfeeding history.

**Table 2:** Prevalence of Reproductive Risk Factors of Female Kaiser Permanente Members Aged 40–65 In Marin County and East/Central Costa County, California

	Marin		East/Central Contra Costa		p-value
			
	Base No.	Percent (95% CI)	Base No.	Percent (95% CI)	
**Menstrual history**					
Age at onset of menarche	415				p = .91
< 12 yrs		17.0 (13.4 – 20.7)		17.1 (13.1 – 21.1)	
12–13 yrs		61.5 (56.7 – 66.2)		62.7 (57.5 – 67.8)	
≥ 14 yrs		21.5 (17.5 – 25.5)		20.2 (16.0 – 24.4)	
Current Menstrual Status	414		347		p = .16
Pre-menopausal		28.1 (23.6 – 47.1)		34.3 (29.2 – 39.5)	p = .07
Peri-menopausal		14.2 (10.8 – 17.5)		14.5 (10.7 – 18.3)	p = .89
Post-menopausal (any reason)		57.7 (52.9 – 62.5)		51.1 (45.8 – 56.5)	p = .08
Natural menopause		47.8 (42.9 – 52.6)		36.2 (31.1 – 41.3)	p = .002
Surgical menopause		9.6 (6.9 – 12.8)		14.9 (11.2 – 18.7)	p = .03
Age (mean) at menopause	217	48.4 yr (se = 0.4)	175	47.3 (se = 0.5)	p = .09
					
**Contraceptive and HRT Use**					
Ever used oral contraceptives	426	84.8 (81.4 – 88.2)	361	81.6 (77.5 – 85.6)	p = .23
Ever used HRT for menopause	423	39.3 (35.1 – 44.6)	358	43.6 (38.3 – 58.9)	p = .32
					
**Pregnancy History**	422		361		p = .12
Never pregnant		14.8 (11.4 – 18.2)		12.2 (8.8 – 15.7)	
One pregnancy		16.7 (12.9 – 20.2)		12.8 (9.2 – 16.1)	
≥ 2 pregnancies		68.5 (64.0 – 73.0)		75.0 (70.4 – 79.5)	
					
**Parity**					
Nulliparous^1^	419	29.2 (24.8 – 33.6)	359	15.4 (11.6 – 19.2)	p < .0001
Uniparous		19.3 (15.4 – 23.1)		19.1 (14.9 – 23.2)	
Multiparous		51.5 (46.7 – 56.3)		65.5 (60.6 – 70.6)	p < .0001
					
**Age at First Live Birth**	420		359		p < .0001
<20 yrs		7.1 (4.6 – 9.6)		9.2 (6.3 – 12.2)	
20–24 yrs		17.9 (14.2 – 21.5)		24.9 (20.5 – 29.6)	
25–29 yrs		22.3 (18.3 – 26.3)		24.9 (20.4 – 29.5)	
No live birth before age 30 ^2^		52.7 (47.9 – 57.5)		40.8 (35.6 – 46.0)	p = .0011
					
**Total months of breastfeeding**	419		360		p = .45
0 (includes no live births)		43.0 (38.2 – 47.7)		37.3 (32.2 – 42.4)	
1–12		30.2 (25.7 – 34.6)		34.1 (29.1 – 39.1)	
13–24		16.3 (12.7 – 19.8)		17.6 (13.6 – 21.7)	
>24		10.5 (7.6 – 13.5)		10.9 (7.6 – 14.3)	
					
**High risk Gail Score **(≥ 1.7) ^3^	403	24.9 (20.7 – 29.1)	347	20.9 (16.7 – 25.2)	p = .17

^1 ^Includes women who were never pregnant or never had a live birth

^2 ^Includes women who never had a live birth

^3 ^Calculated only for women with no history of breast cancer

Further analysis of reproductive responses indicated some additional similarities and differences. The percentage of women who had never been pregnant was similar (14.8% vs. 12.2%, p = 0.12). Among women who had experienced at least one pregnancy, a significantly higher percentage of MC than ECCC women reported more pregnancies than births (57.3% vs. 46.4%, p = .006). Among those women who had borne at least one child, there was no significant difference between groups in percentage who had given birth by age 30 (66.8% vs. 70.0%, p = .41).

The groups did not differ significantly on percentages at above average risk for invasive breast cancer based on Gail risk scores (calculated for women with no history of breast cancer). When we created an adapted Gail variable that combined women with a score ≥1.7 and those with a history of breast cancer into a modified high risk category, there were still no significant differences between the MC and ECCC groups [modified high risk: 28.7%; (95% CI 22.9–30.4) vs. 24.5% (95% CI 20.1–29.0)]. These results did not change when adjusting for age (results not shown).

Table 3 shows that the two groups were comparable with regard to weekly moderate or strenuous physical activity and BMI category. Compared to ECCC women, a slightly higher percentage of MC women consumed more than one drink per day (i.e., estimated consumption averaging > 7 alcoholic drinks a week, 21.5% vs. 16.1%, p = .06), and a significantly lower percentage of MC women were abstainers or very infrequent drinkers (34.4% vs. 41.9%, p < .05). However, heavy drinking (>14 drinks/week) was extremely low in both groups. While the groups did not differ in current smoking, MC women were also significantly more likely than ECCC women to be former smokers (43.6% vs. 31.2%, p = 0.0004) and less likely to be never smokers (48.3% vs. 61.7%, p = 0.0002). Both groups had similarly high (89%) percentages of women who reported having had a mammogram within the past two years, and the percentages of women who reported never have been screened were similarly very low.

**Table 3:** Prevalence of Behavioral Risk Factors and Breast Cancer Screening Among Female Kaiser Permanente Members Aged 40–65 in Marin County and East/Central Costa County, California

	Marin		East/Central Contra Costa		p-value
			
	Base No.	Percent (95% CI)	Base No.	Percent (95% CI)	
**Smoking status**	426		361		
Never smoked		48.3 (43.5 – 53.1)		61.7 (56.6 – 66.8)	p = .0002
Former smoker		43.6 (38.8 – 48.3)		31.2 (26.3 – 36.0)	p = .0004
Current smoker		7.7 (5.1 – 10.3)		7.2 (4.5 – 10.0)	p = .80
Occasional		3.1 (1.4 – 4.8)		2.3 (0.7 – 3.9)	
Daily		4.9 (2.9 – 7.0)		4.9 (2.6 – 7.2)	
					
**Alcohol use in past 12 mos.**	417		360		
Never or <1/month		34.4 (29.8 – 39.0)		41.9 (36.7 – 47.0)	p < .05
Averaged > 7 drinks/week		21.5 (17.5 – 25.4)		16.1 (12.3 – 19.9)	p = .062
Averaged > 14 drinks/week		4.2 (2.3 – 6.0)		2.7 (1.0 – 4.4)	p = .30
					
**Exercise**					
**Usual # days of moderate or strenuous exercise**	426		362		p = .13
< 1 day/week		19.4 (15.6 – 23.2)		23.3 (18.9 – 27.7)	
1–2 days/week		11.9 (8.8 – 15.1)		15.8 (11.9 – 19.6)	
3–4 days/week		23.9 (19.8 – 27.9)		19.3 (15.2 – 23.4)	
≥ 5 days/week		44.8 (40.0 – 49.5)		41.6 (36.5 – 46.8)	
**Usual # days of ≥30 min of moderate/strenuous exercise**	426		362		p = .16
< 1 day/week		31.1 (26.7 – 35.6)		37.7 (32.6 – 42.7)	
1–2 days/week		11.6 (8.4 – 14.6)		13.2 (9.7 – 16.8)	
3–4 days/week		20.7 (16.8 – 24.6)		18.1 (14.0 – 22.1)	
≥ 5 days/week		36.6 (32.0 – 41.2)		31.0 (26.1 – 35.9)	
					
**BMI**	424		357		p = .50
% Underwt (BMI 14 – < 18.5)		2.7 (1.1 – 4.3)		3.6 (1.7 – 5.6)	
% Normal-Overwt (BMI 18.5–<30)		81.5 (77.8 – 85.3)		78.4 (74.0 – 82.7)	
% Obese (BMI ≥ 30)		15.7 (10.3 – 19.2)		18.0 (14.0 – 22.0)	
					
**Mammogram (ages 41–65)**	418		346		p = .70
In last 2 years		88.5 (85.4 – 91.7)		88.6 (85.1 – 92.0)	
> 2 years ago		8.2 (5.5 – 10.8)		9.1 (6.0 – 12.2)	
Never		3.3 (1.4 – 5.1)		2.3 (0.6 – 4.1)	

Breast cancer incidence rates for KPNC female members aged 40–64 in the MC and ECCC comparison area for the 5-year period 2000–2004 are shown in Table 4. The age-adjusted rate and ethnicity-adjusted rates were approximately 7% higher for MC women than ECCC women and the absolute difference is approximately 17 cases per 100,000, though these differences were not statistically significant.

**Table 4:** Five-Year (2000–2004) Incidence Rates of Invasive Breast Cancer for Kaiser Permanente Female Members Aged 40–64 in Marin County and East/Central Contra Costa County, California^1^

	Marin	East/Central Contra Costa	p-value
**Number of cases/Population**	184/65,937	384/148,388	
**Five-year rate per 100,000 women adjusted for age**	257.7 (220.9 – 298.8)	240.5 (216.8 – 266.0)	p = .69
**Five-year rate per 100,000 women adjusted for age and ethnicity**	264.7 (224.6 – 309.8)	245.7 (218.9 – 274.9)	p = .74

^1 ^Rates adjusted to the age and race/ethnic (White nonHispanic vs. Other) composition of the female population in each geographic area per U.S. Census 2000 estimates.

## Discussion

This study compared the breast cancer risk factor prevalence among a randomly selected group of female KPNC members in Marin County, CA, which has a historically high breast cancer incidence rate, with risk factors among a group of KPNC members in a geographically separate but sociodemographically similar area in nearby Contra Costa County. The study design enabled the comparison of established risks and incidence rates while minimizing potential confounding effects of sociodemographic characteristics associated with breast cancer risk (race, educational attainment, and income). The results show that matching the KPNC Marin County women with a sociodemographically similar comparison member group outside Marin County substantially diminished differences in breast cancer incidence and prevalence of many, but not all, breast cancer risk factors.

The reasons for the documented association between SES and breast cancer incidence is not well understood, but it has been suggested that SES acts as a marker for such factors as screening and reproductive behaviors [18] and childhood nutrition [19]. A recent study of women in Norway and Sweden examined whether an association between overall breast cancer risk and SES persists after simultaneously controlling for more known risk factors [20]. The authors found that controlling for parity, age at first birth, BMI, height, age at menarche, age at menopause, alcohol use, and use of hormonal contraceptives eliminated the relationship between educational level and breast cancer risk. Our study similarly showed that matching a known high risk population with a sociodemographically (race/ethnicity, income, and education) comparable population resulted in comparable prevalence of many breast cancer risk factors (i.e., family history of breast cancer, physical activity, BMI, age at menarche, oral contraceptive and HRT usage, and duration of breastfeeding), but did not remove differences in potentially important reproductive, behavioral, and genetic risk factors.

The most salient differences between the women from the high breast cancer incidence county and SES-matched comparison group were in reproductive factors. The Marin County women were less likely to have given birth before age 30 and more likely to be nulliparous. While previous studies have calculated that approximately 30% of breast cancer cases are attributable to late age at first birth/nulliparity [21], the two populations in this study did not have significantly different breast cancer incidence rates. This could be due to differences between the sample used in calculating risk factor prevalence and the population for which rates were calculated or to differences in how these factors operate in the presence of the other combinations of risk factors in these populations. While the Marin County women were also more likely than the comparison women to have a greater number of pregnancies than live births, we did not determine whether pregnancy losses were spontaneous or induced. Recent studies have shown no increase in breast cancer risk for women with a history of either spontaneous or induced pregnancy loss [22,23]. Further exploration of breast cancer risk associated with nulliparity and late age at first birth, incorporating hormonal and genomic indicators, is needed [24].

Smoking history and alcohol consumption differed between the Marin County and the comparison group. While both groups had current smoking rates well below the 2004 State of California prevalence of 14.6% [25], the Marin County women were significantly more likely than the comparison group to be former smokers and significantly less likely to be never smokers. Marin County women were significantly less likely than the comparison women to abstain from alcohol or drink less than 1 drink per month, and more likely to consume more than 1 drink per day. Heavy drinking was extremely low among both groups.

These differences in smoking history and alcohol consumption could help explain part of any increased incidence of breast cancer in Marin County. While the association between smoking and breast cancer incidence is inconsistent in the literature, a significant association has been found in large studies between one drink per day and breast cancer. For example, based on reanalysis of data from 53 international studies, Hamajima and colleagues estimated that every additional drink per day increases the relative risk of breast cancer by 7.1%, and that 4% of breast cancer cases in developed countries could be attributed to alcohol if alcohol is causally related to breast cancer [26]. While that study found little independent effect of smoking on breast cancer incidence, another reanalysis of data from 10 studies found that smoking is related to breast cancer in women with a certain genotype, particularly N-acetyltransferase 2 (NAT2) slow acetylation genotype, which the authors note is present in at least one-half of Caucasian women [27]. Alcohol and tobacco use have been found to be important breast cancer risk factors in Marin County in the past. In particular, a matched case-control study conducted within Marin County found an almost a four-fold increased odds of breast cancer in women who consumed on average greater than three drinks per day and a 2.4-fold association with greater than 28.5 pack-years of tobacco consumption [7].

Women in Marin County may be more likely to have genetic risks associated with Ashkenazi ethnicity [28,29], but larger studies are needed to confirm this. However, despite the differences in age, race/ethnicity, and reproductive risk factors, the groups did not significantly differ on percentages of women at high risk for developing breast cancer based on the Gail composite risk factor score.

Since this study essentially matched KPNC members from Marin and the comparison community on race-ethnicity, income, and education at the outset, the findings of similar breast cancer risk factor profiles and statistically comparable cumulative breast cancer incidence rates among KP members residing in the two areas provide some evidence for what some earlier authors have suggested, i.e., that regional differences in breast cancer rates in the SFBA may be due to regional differences in SES factors [30]. These findings suggest that future comparisons of breast cancer incidence rates between geographic areas should attempt to adjust for population differences in income and education in addition to age and race/ethnicity.

The restriction of the study to female KPNC health plan members for this pilot study had several advantages. Differences between groups with regard to access to care, model of health care delivery, and exposure to breast cancer prevention measures, including screening and risk factor education, were minimized. In addition, breast cancer incidence rates could be calculated using counts of the female membership. However, there are several potential limitations to the study design that could affect interpretation of the results. Our study power was not high enough to detect differences in breast cancer risk factors of less than ten percentage points due to a lower than anticipated response rate. Additionally, if the substantially higher survey response for the Marin County member sample is due to high level of public awareness about elevated breast cancer incidence rates in Marin County created by local media and advocacy groups, this might have introduced response bias. Because KPNC, like most health plans, did not have computerized information about the race/ethnicity of its members, we had to rely on survey estimates to create the ethnicity-adjusted denominators in breast cancer incidence measures. Finally, we do not know how generalizable these results are to the general populations of the two study communities. Data from the 2003 California Health Interview Survey suggest that overall, the KPNC membership has lower percentages of adults at the lower and upper extremes of household income than both the general and privately insured populations in Northern California, and that while the membership is very similar to the privately insured population with regard to educational attainment and health-related behaviors, it is much better educated and has a lower prevalence of behavioral health risks than the general population [31]. If the segment of the population not covered by KPNC had substantially different rates of breast cancer risks and breast cancer, the difference in the KPNC coverage rate for Marin County and the East/Central Contra Costa County comparison area (28% vs. 38%, respectively) might not be representative of the difference in breast cancer rates in the general populations of the two communities.

## Conclusion

In these sociodemographically matched health plan member populations, we found statistically comparable breast cancer incidence rates and similar prevalence of many, but not all, established breast cancer risk factors. The effects of reproductive risks factors, Ashkenazi Jewish heritage, smoking history, and alcohol consumption with regard to breast cancer risk in Marin County should be further evaluated in larger and more representative community samples. When possible, future comparisons of breast cancer incidence rates between regions should adjust for differences in income and education in addition to age and race/ethnicity, preferably by using a sociodemographically similar comparison group.

## Abbreviations

SES: socioeconomic status; MC: Marin County; ECCC: East/Central Contra Costa County; CA: California; KPNC: Kaiser Permanente Medical Care Program in Northern California; BMI: body mass index; HRT: hormone replacement therapy.

## Competing interests

The authors declare that they have no competing interests.

## Authors' contributions

SL and NG conceived the study. SL, NG, LP, and RE collaborated on the study design and survey instrument; NG secured funding for the study and her team collected and cleaned all survey data. SL, NG, LP, and RE structured the statistical analyses, and NG and MP analyzed the survey data. CU conducted the analyses comparing breast cancer incidence rates in the two communities. SL, NG, LP, RE, and MP interpreted the results of the analyses and collaborated on writing the manuscript.

## Pre-publication history

The pre-publication history for this paper can be accessed here:

http://www.biomedcentral.com/1472-6874/9/6/prepub

## Supplementary Material

Additional file 1 Survey questionnaire used for the study. Click here for file
