# Supplementary material for: Differences in reproductive risk factors for breast cancer in middle-aged women in Marin County, California and a sociodemographically similar area of Northern California
Source: BMC Womens Health. 2009 Mar 25;9:6. doi: 10.1186/1472-6874-9-6 (PMC2670264; doi:10.1186/1472-6874-9-6)
Supplement: Additional file 1 — Survey questionnaire used for the study. [file 1472-6874-9-6-S1.pdf]

Kaiser Permanente Women's Health Risk Factor Survey

Address Corrections (Please **print**)

____

____

Daytime phone: (____) ____________

This survey will help both Kaiser Permanente and California public health departments learn whether differences in breast cancer rates in communities may be explained by differences in generally known breast cancer risks among community residents.

The survey is **confidential.** All reports using survey information will describe whole groups of members, not individuals, and your name will never be included in any reports. Your answers will not be shared with your doctor or employer, become part of your medical record, or affect your medical care, Health Plan membership, or dues.

If you have any questions about or need help with the survey, call us toll-free at (1-800) 723-8055 (select "Women's Health Risk Factor Survey") or email us at WHRFS@dor.kaiser.org. Please write your phone number and any address corrections above**.** **<u>This questionnaire should be filled out ONLY for the person named above</u>.**

Please return your questionnaire to: Kaiser Permanente, Division of Research, P.O. Box 12916, Oakland, CA 94604. A pre-addressed, postage paid envelope has been enclosed for this purpose.

Thank you for your help.

FAMILY HISTORY

1. Please check the box of any **female blood relatives** who have been diagnosed with breast cancer. *(Blood/biological relative = related by birth, not adopted or related by marriage; Maternal=from mother's side of the family, Paternal = from father's side of the family)*

   ❒ Biological daughter(s) – How many? ___
   ❒ Biological (birth) mother
   ❒ Biological sister(s) – How many?___
   ❒ Maternal aunt
   ❒ Paternal aunt
   ❒ Maternal grandmother
   ❒ Paternal grandmother
   ❒ Maternal female cousin(s)
   ❒ Paternal female cousin(s)
   ❒ No female blood relatives had breast cancer
   ❒ I was adopted

   1a. Please put a star (*) next to the box of any of these relatives who developed breast cancer before age 40.

BREAST HEALTH HISTORY

2. Have you ever had a breast biopsy to check for cancer?

   ❒ No
   ❒ Yes → ***Please answer 2a-2b***

   2a. How many breast biopsies have you had? ____ Biopsies

   2b. What were the diagnoses: *(Check all that apply)*

   ❒ Invasive breast cancer
   ❒ Ductal carcinoma in-situ (DCIS)
   ❒ Lobular carcinoma in situ (LCIS)
   ❒ Atypical hyperplasia
   ❒ Fibrocystic disease, other
   ❒ Not sure

3. Have you ever been told by a doctor that you had breast cancer?

   ❒ No
   ❒ Yes → How old were you when you were first diagnosed with breast cancer? ____ Years Old

REPRODUCTIVE HISTORY

4. How old were you when you had your first menstrual period? ………………………… ____ Years Old

5. How many times in total have you been pregnant? *(If never, please write "0")* …….. ____ Times
   If "0" times, skip to Question 7

   5a. How many live births have you had?
   (A multiple birth is considered one birth. If none, write" 0") ……………………… ____ Live Births
   If "0" times, skip to Question 7

   5b. How old were you at the time of your first live birth? ……………………………… ____ Years Old

6. Did you breastfeed any of your children?

   ❒ No or Not applicable
   ❒ Yes → ***Please answer 6a and 6b***

   6a. How old were you when you first breastfed? ……………………….. ____ Years Old

   6b. Please add together the number of months you breastfed each of your children *(Consider a multiple birth as one child).*
   How many months in total have you breastfed? …………………… ____ Months

7. Have you ever taken birth control pills for one month or longer?

❒ No
❒ Yes → ***Please answer 7a - 7c***

7a How old were you when you first used birth control pills? ___ Years Old

7b. How old were you when you last used birth control pills? ___ Years Old

7c. How long in total have you used birth control pills?
(Do not include times when you temporarily stopped) ___ Years ___ Months

8. Have you ever used prescription female hormones or hormone replacement therapy (HRT) like Prempro, Premphase, or Premarin for symptoms of menopause or for other reasons? *(Do not include use of birth control pills, creams, vaginal suppositories, or dietary supplements)*

❒ No
❒ Yes → ***Please answer 8a - 8e***

8a. How old were you when you started using female hormones or HRT?
(not oral contraceptives) ………………………………………………… ___ Years Old

8b. How old were you when you stopped using female hormones or HRT? ___ Years Old

8c. About how long in total did you use female hormones or HRT?
(Do not include times when you temporarily stopped) ……… ___ Years ___ Months

8d. About how long did you take HRT containing only estrogen
(such as Premarin)? …………………………………………… ___ Years ___ Months

8e. About how long did you take HRT containing progestin and estrogen together (such as Prempro, Premphase, or Premarin with progesterone)? ………………………………… ___ Years ___ Months

9. What describes your menopausal status?

❒ Pre-menopausal (my menstrual periods still follow a cycle)
❒ Peri-menopausal (there have been significant changes in how often or how long my menstrual periods are, or I have not had a menstrual period for 3 to 11 months)
❒ My menstrual periods stopped after a hysterectomy (to remove uterus) or oophorectomy (to remove ovaries) -- I was still having menstrual periods before the surgery)
↳ At what age did you have the hysterectomy or oophorectomy? …… ___ Years Old
❒ Post-menopausal -- I have not had a natural (not caused by medication) spontaneous menstrual period for 12 months or more)
↳ At what age did you have your last natural menstrual period? ……… ___ Years Old

❒ Other (describe):____

LIFESTYLE AND HEALTH-RELATED PRACTICES

10. When did you last have a:	**Within past 12 months**	**1 to 2 years ago**	**2 to 3 years ago**	**More than 3 years ago**	**Never**
a. A mammogram?	❒	❒	❒	❒	❒
b. A breast exam by a doctor or nurse?	❒	❒	❒	❒	❒

11. Was your most recent mammogram done at a Kaiser medical facility?

❒ No ❒ Yes ❒ Don't know ❒ Does not apply

12. How tall are you without shoes? ……………………………………… ____ Feet ____ Inches

13. How much do you weigh without your shoes and clothes? ………… ____ Pounds

14. Do you smoke cigarettes?

❒ Never smoked ❒ Used to smoke, but quit ❒ Smoke occasionally ❒ Smoke daily

15. During the past 30 days, how many days per week did you have any drinks containing alcohol, on average? ***(If none, write "0" and skip to Question 17)*** ____ Days per Week

16. On the days when you had a drink, about how many drinks did you have, on average? *(1 drink = 12 oz. of beer, 4 oz. of wine, or 1 oz. of hard liquor)* .... ____ Drinks

17. During the past 12 months, how often have you usually had a drink containing alcohol?

❒ Every day
❒ 6 days a week
❒ 5 days a week
❒ 4 days a week
❒ 3 days a week
❒ 1 to 2 days a week
❒ 2 to 4 days a month
❒ 1 day a month or less
❒ Never → ***Please answer 17a and 17b***

If you checked "Never", please answer 17a and 17b:

17a. How long ago did you stop having drinks containing alcohol? ___ Years ___ Months Ago
(If you never drank at all as an adult, check this box ❒ and skip to Question 19)

17b. Before you stopped, how often did you usually have drinks containing alcohol? .... ___ Days per Week **or** ___ Days per Month

18. On the days when you had [or you used to have] a drink, how many drinks did you usually have? *(1 drink = 12 oz. of beer, 4 oz. of wine, or 1 oz. of hard liquor)* .... ____ Drinks

19. During the past 12 months, how often did you usually participate in STRENUOUS physical activities (such as aerobics, calisthenics, running, jogging, basketball, cycling on hills, swimming laps)?

❒ Every day
❒ 6 days a week
❒ 5 days a week
❒ 4 days a week
❒ 3 days a week
❒ 2 days a week
❒ 1 day a week
❒ 2 to 4 times a month
❒ Once a month or less
❒ Never → ***Skip to Question 20***

19a. On those days when you participated in STRENUOUS physical activities, how many total minutes of STRENUOUS exercise did you get per day, on average? *(1 hour = 60 minutes)*

❒ Over 120 minutes
❒ 91 to 120 minutes
❒ 61 to 90 minutes
❒ 46 to 60 minutes
❒ 31 to 45 minutes
❒ 16 to 30 minutes
❒ 10 to 15 minutes
❒ Less than 10 minutes

20. During the past 12 months, how often did you usually participate in MODERATE physical activities (such as brisk walking, walking up hills, volleyball, cycling on level streets, tennis, or dancing)?

❒ Every day
❒ 6 days a week
❒ 5 days a week
❒ 4 days a week
❒ 3 days a week
❒ 2 days a week
❒ 1 day a week
❒ 2 to 4 times a month
❒ Once a month or less
❒ Never → ***Skip to Question 21***

20a. On those days when you participated in MODERATE physical activities, how many total minutes of MODERATE exercise did you get per day, on average? *(1 hour = 60 minutes)*

❒ Over 120 minutes
❒ 91 to 120 minutes
❒ 61 to 90 minutes
❒ 46 to 60 minutes
❒ 31 to 45 minutes
❒ 16 to 30 minutes
❒ 10 to 15 minutes
❒ Less than 10 minutes

21. How does the amount of exercise you get now compare with how much you got 3 years ago?

a. Strenuous exercise: ❒ Get more now ❒ Get about the same now ❒ Get less now

b. Moderate exercise: ❒ Get more now ❒ Get about the same now ❒ Get less now

PERSONAL CHARACTERISTICS

22. What is your age? _____ Years Old

23. What describes your race and ethnicity?
Check ALL that apply. If you check more than one racial/ethnic background, please also put a star (*) next to the category with which you most closely identify.

❒ White or European-American
❒ African-American
❒ Other Black *(specify)*:____________________
❒ Mexican or Central American ancestry
❒ Other Hispanic/Latino *(specify)*:_____________
❒ Middle Eastern (Arab, Palestinian, Israeli sabra)
❒ South Asian (e.g., Indian or Pakistani)
❒ Chinese
❒ Southeast *Asian (specify)*:_____________
❒ Japanese
❒ Korean
❒ Filipino
❒ Other *Asian (specify)*:________________
❒ Hawaiian/Pacific Islander *(specify)*:______
❒ Native American Indian or Alaska Native
❒ Other *(specify)*:_____________________

24. Were one or more of your mother's or father's biological parents (not step-parents) an Ashkenazi (Northern, Central, or Eastern European) Jew?

❒ No ❒ Yes ❒ Not sure

25. What is the highest level of school you completed? *(Check only ONE answer)*

❒ 8th grade or less
❒ 9th - 11th grade
❒ 12th grade (high school graduate or G.E.D.)
❒ Some college or technical school
❒ Completed 4-year college (B.A., B.S., etc.)
❒ Completed graduate degree

26. Which of the following best describes your total household (family) income from all sources in 2002, before taxes? *(Check only ONE answer)*

❒ Under $15,000
❒ $15,000 - $25,000
❒ $25,001 - $35,000
❒ $35,001 - $50,000
❒ $50,001 - $65,000
❒ $65,001 - $80,000
❒ $65,001 - $80,000
❒ $80,001 - $100,000
❒ $100,001 - $150,000
❒ $150,001 -$200,000
❒ Over $200,000

26a. How many people were supported by this income? ____ Persons

27. How many total years have you lived in Marin County? ____ Years

28. In what other California counties have you lived since you were 25 years old?

Name of California County	Years in Which You Lived in this County
________________________	________ to _________
________________________	________ to _________
________________________	________ to _________

This is the end of the survey. Thank you for your help.
